# Supplementary material for: Socioeconomic disparities in anthropometric status among primary school children: A potential association with school meals
Source: PLoS One. 2025 Apr 2;20(4):e0321289. doi: 10.1371/journal.pone.0321289 (PMC11964276; doi:10.1371/journal.pone.0321289)
Supplement: Attachment — Submitted filename: Response to Reviewers.docx [file pone.0321289.s003.docx]

# Response to reviewers

We sincerely appreciate the time and effort of the academic editor and reviewers in evaluating our manuscript.

# Reviewer #1

**Reviewer comment: The authors have not categorically pointed out the inculsion and exclusion crieteria for the subject sample.**

**Response**: We appreciate the reviewer's observation. The inclusion criteria are implicit in the *Materials and methods* section, where we describe **selecting** a **random sample of healthy children aged 5–10 years** from primary schools and **excluding** those with **chronic health conditions or uncertain dates of birth**. However, to address the reviewer’s concern, we have now explicitly listed them in the revised text.

**Revised text**: 'Inclusion criteria were healthy primary school children aged 5–10 years, while children with chronic health conditions or uncertain birth dates were excluded.'

**Reviewer comment: Sample size (506) does not represent the socioeconomic disparities associated with growth and nutritional status of the children at national level hence a larger study is needed to depict if the same situation prevail in the country.**

**Response**: We appreciate the reviewer’s comment. However, our study **does not claim to represent the entire country**, as is evident from the title and stated aim. The study specifically focuses on primary school children **in Wad-Madani City** to provide localized insights into socioeconomic disparities in growth and nutritional status. However, as a side note, given the similar circumstances and population structure across many regions in Sudan, we believe there are no strong reasons why these findings may not cautiously apply to other similar settings in the country

**Reviewer comment:** **There have been spelling and grammar mistakes in the text which need to be addressed.**

**Response:** We appreciate the reviewer's feedback. The manuscript has undergone thorough proofreading, and we believe any language issues are minimal. However, we have carefully rechecked the text and made refinements where necessary to ensure correctness.

**Reviewer comment: The verbatin while interpreting data has to be avoided as it creats a bit confusion for the readers implying the article should have more clarity.**

**Response:** We thank the reviewer for their comment. However, the concern is not entirely clear. If the issue pertains to the clarity of data interpretation, we have carefully reviewed and refined relevant sections to ensure that our explanations are precise and unambiguous.

# Reviewer #2

**Reviewer comment: Growth should be expunged in the title as direct growth assessment was not conducted. The growth indices assessed depict nutritional status.**

**Response**: We sincerely appreciate the reviewer's comment. However, we respectfully disagree with the suggestion to remove 'growth' from the title for the following reasons:

- **Our study, certainly, assessed growth using standard anthropometric measures:**
  - We measured **height, weight, and BMI** and **plotted them** on established **growth** charts, which is the **standard** method for **assessing** **one-point growth status** in children.
  - This is the same approach used in clinical practice and epidemiological studies to assess child **growth** (<https://www.cdc.gov/growthcharts/background.htm>).
- **Nutritional status was assessed as a reflection of growth assessment:**
  - We could **not** have assessed nutritional status **without** first **assessing** **growth** through these standard anthropometric indices.
  - Both assessments were conducted **at a single point in time**, aligning with widely accepted methodologies (<https://www.ncbi.nlm.nih.gov/books/NBK537315>).
- **Cross-sectional (one-off) growth assessments are an established method in child health research:**
  - The **CDC growth charts**, one of the most widely used references, were developed using this exact approach—measuring **different** children’s growth at different ages **rather than tracking** growth velocity over time.
  - It is common practice in both **clinical** and **public health research** to use **single-time-point growth assessments** to evaluate children's health status (<https://pubmed.ncbi.nlm.nih.gov/12043359>).
- **We used 'growth charts,' not 'nutritional status charts.'**
  - The charts and Z-score standards we used are called **'growth** charts**,'** not 'nutritional status charts.'
  - Removing 'growth' would inaccurately reflect the **standard terminology used in growth and nutritional research** (<https://www.who.int/tools/child-growth-standards/standards>).
- **Title relevance and study visibility:**
  - Keeping 'growth' in the title is essential for clarity, accuracy, and search **visibility** in scientific literature.
  - It correctly represents the study’s focus and ensures it is **easily identified by relevant readers and researchers**.
- **Clarification on what we did not assess:**
  - We did **not** assess **longitudinal growth velocity** (growth rate over time), which seems to be the reviewer's concern.
  - However, **cross-sectional** growth assessments remain the primary method for **evaluating child growth and nutritional status in epidemiological studies** (<https://www.cdc.gov/growth-chart-training/hcp/using-growth-charts/who-methodology.html>).

**Given these points, we respectfully request that the reviewer reconsider this suggestion and allow us to retain 'growth' in the title and relevant sections.**

**Reviewer comment: Abstract: The method section of the abstract is not detailed enough to show how the objective was achieved. Was comparison of the Z scores between private and public schools the only assessment conducted? The study design, sample size and sampling techniques used are missing. Which data collection methods were employed? How were the data analysed? The title showed that the authors also assessed growth. How was this conducted?**

**Response:** We thank the reviewer for her valuable comments. We have **revised the abstract to provide additional methodological details**, including the study design, sample size, sampling method, data collection approach, and statistical analyses.

Regarding the reviewer's last point, we have already clarified in our previous response that growth was assessed using standard anthropometric measures (height, weight, and BMI), which were **plotted** on WHO **growth charts**. This remains the standard approach for evaluating growth at a single point in time in both clinical and epidemiological settings.

We appreciate the reviewer’s feedback and believe the revised abstract now fully addresses the concerns raised.

**Revised abstract:**
**Objective.** To assess the growth and nutritional status of children in primary schools across different socioeconomic groups in Wad-Madani City, Central Sudan, and map it to World Health Organization (WHO) standards; and to investigate a potential association between school meal intake and nutritional status.

**Methods.** This **cross-sectional** anthropometric **study** involved a randomly selected sample of 506 children from 10 primary schools in the city. **Height and weight were measured following WHO standards and converted into** Z-scores for weight-for-age (WAZ), height-for-age (HAZ), and BMI-for-age (BAZ). We compared the mean Z-scores between children in the private and public school sectors, adjusting for ethnicity and other potential predictors. **Statistical analyses included multivariate linear regression to assess predictors of growth and nutritional status, alongside group comparisons using appropriate statistical tests.**

**Results.** Children in public schools had significantly lower BAZ and HAZ levels compared to both WHO standards and private school children. The mean BAZ was -1.0 (SD = 1.23) for public school children and -0.13 (SD = 1.40) for private school children (p = 0.001), with 17.8% (n = 57) of public school children classified as thin (wasted) or severely wasted. The median HAZ was -0.20 (95% CI: -0.34, -0.02) for public school children and 0.19 (95% CI: 0.03, 0.40) for private school children (p < 0.001). Additionally, children in suburban public schools had a significantly lower mean HAZ (-0.46, SD = 11.33) compared to those in urban public schools (p = 0.009). **Compared to WHO growth standards, public school children had significantly lower mean WAZ (p < 0.001), HAZ (p = 0.002), and BAZ (p < 0.001).** Children who received school meals had significantly higher WAZ (mean difference = 0.619, p = 0.001), HAZ (mean difference = 0.401, p = 0.010), and BAZ (mean difference = 0.588, p = 0.003) across the entire sample. Even within the public-school subgroup, while statistical significance was not reached, all three parameters—WAZ (mean difference = 0.334, p = 0.074), HAZ (mean difference = 0.262, p = 0.123), and BAZ (mean difference = 0.299, p = 0.132)—remained consistently higher among those who received school meals.

**Conclusion.** Public school children exhibit unfavorable growth and nutritional status, which may be attributed to inadequate nutritional and calorie intake. School meals may improve nutritional outcomes. We propose urgent intervention through the provision of nutritionally adequate school meals.

**Reviewer comment: The results are clear but results on growth is missing.**

**Response**: The abstract has been revised to address this. See the above response please.

**Reviewer comment: By growth, I expected to see baseline and endline values and mean differences that shows increase or decrease in growth parameters (weight, height, BMI). When these parameters are related to age or any other parameter, it becomes an index of nutritional status.**

**Response:** We thank the reviewer for their comment. However, the expectation of “**baseline and endline measurements**” reflects **growth velocity** assessment (**growth rate over time**), which is a **complementary but not primary** method for assessing growth in both clinical and epidemiological settings **in children**. The cross-sectional approach, where anthropometric indices are measured and compared with standardized growth charts **according to age** and sex, remains the most widely practiced method in child growth assessment.

We also respectfully would like to clarify that **in children, all growth parameters must be related to age**; otherwise, growth assessment would **not** be meaningful. No clinically or epidemiologically sound growth assessment is conducted without relating anthropometric measurements to age. This is the basis of all internationally recognized child growth standards (WHO, CDC).

As discussed in our previous response, our study followed this standard cross-sectional methodology, making it an appropriate and widely accepted approach. We hope this clarification addresses the reviewer’s concern.

**Reviewer comment: Growth used in the conclusion is inappropriate because growth was not assessed. The authors assessed nutritional status; the growth indicators are not direct assessment of growth pattern. The key words should also be reviewed in line with this statement.**

**Response:** We thank the reviewer for their comment. However, we believe this concern has already been sufficiently addressed in our previous responses. As previously clarified, our study assessed growth using standard anthropometric indicators (weight-for-age, height-for-age, and BMI-for-age), which are internationally recognized methods for assessing child growth at a single point in time. These indicators are referred to as 'growth indicators' in WHO and CDC classifications and are widely used in both clinical and epidemiological research.

The reviewer's concern likely stems from conflating growth assessment with growth velocity tracking over time, which was not the study’s aim. Cross-sectional growth assessment using standardized Z-scores remains the primary and most widely accepted approach for evaluating child growth in epidemiological studies.

Given this, we believe that the term ‘growth’ remains appropriate in the conclusion and keywords.

**Reviewer comment: weight for height or body mass index (BMI) for age: these are indices and should be written as compound words.**

**Response:** We thank the reviewer for their observation. Indeed, 'weight-for-height' and 'BMI-for-age' are widely accepted terms in scientific literature and are typically written with hyphens **when used as compound adjectives** (e.g., *weight-for-height Z-score* or *BMI-for-age reference standard*). **When used as standalone terms** (e.g., *weight for height is an indicator of acute malnutrition*), hyphens are **not** required. This convention follows the standard usage in WHO and CDC publications, where 'weight-for-height' and 'BMI-for-age' are written with hyphens when modifying a noun but not necessarily when used independently (please refer to the following content to verify our response: [WHO Growth Standards](https://www.who.int/tools/child-growth-standards) and [CDC Growth Chart Training](https://www.cdc.gov/growth-chart-training/hcp/using-growth-charts/who-methodology.html)).

**Revised text**: We have reviewed the manuscript to ensure consistent usage of these terms following standard scientific conventions.

**Reviewer comment: We estimated a sample size of 392: This statement is inappropriate. The authors estimated the sample size instead of calculating it based on a prevalence of malnutrition of 15%, a confidence limit of 5%, a cluster effect of 2 and a 95% confidence level. Is there any explanation for this?**

**Response:** We thank the reviewer for their observation. We acknowledge that 'calculated' may be a more precise term than 'estimated' in this context. The minimum sample size was determined using a prevalence of malnutrition of 15%, a confidence limit of 5%, a cluster effect of 2, and a 95% confidence level. To enhance clarity, we have revised the wording in the manuscript to reflect this.

**Revised text:** With a population of around 90000 primary school children in Wad-Madani, a hypothesized prevalence of malnutrition of 15% [10], a confidence limit of 5% and, a cluster effect of 2, we calculated a minimum required sample size of 392 for a 95% confidence level.

**Reviewer comment: The authors estimated a sample size of 392 but distributed 1000 consent forms. What was the essence of estimating (uncalculated) the sample size. It appears that selection of the pupils for the study was dependent on return of consent forms. It is also not clear how they arrived at the 1000 pupils given the forms.**

**Response:** We thank the reviewer for their comment. The calculated sample size of 392 represents the **minimum** required number of participants to achieve sufficient statistical power. However, we aimed for a **higher number** to account for potential non-response and missing data. It is common practice to **recruit more participants** to account for non-response, withdrawals, or missing data.

To maximize recruitment, we distributed 1000 consent forms, anticipating that some parents would not return them. This approach aligns with best practices in epidemiological and school-based research, where **oversampling is commonly used to ensure adequate recruitment** (<https://pmc.ncbi.nlm.nih.gov/articles/PMC5508166/?utm_source=chatgpt.com>)**.**

Selection was therefore dependent on the return of consent forms, as is standard in school-based studies requiring parental approval (<https://bmjopen.bmj.com/content/13/6/e070277>). We have clarified this in the manuscript to ensure transparency.

**Revised text:** With a population of around 90000 primary school children in Wad-Madani, a hypothesized prevalence of malnutrition of 15% [10], a confidence limit of 5% and, a cluster effect of 2, we calculated a minimum required sample size of 392 for a 95% confidence level.

**Reviewer comment: How many children were selected from each of the areas (public/private) schools and on what basis. Same applies to each of the schools.**

**Response**: We thank the reviewer for this comment. The manuscript provides details on the total number of children selected from public and private schools, as well as their distribution across urban and suburban areas. However, data on the exact number of participants from each of the 10 schools was **not** collected. Selection within each school **was not based on proportional allocation** to school enrollment size but rather on a **random selection of children aged 5–10 years** from the list of eligible students in the participating schools.

We have revised the manuscript to explicitly state that the selection within each school was not proportionate to school enrollment size.

**Revised text**: **Selection within each school was not proportionate to school enrollment size; instead, an equal opportunity approach was applied to ensure feasibility in recruitment across schools.**

**Reviewer comment:** **A random sample of healthy children aged 5 and 10 years was selected from each school: How did the authors establish that the children were healthy? There are different types of random sampling. The authors should describe the ones used. Sampling technique is an important aspect of research. The authors should detail step by step how the sample was selected.**

**Response:** We thank the reviewer for this comment. The selection of children was done using **simple** random sampling from a list of enrolled students within the eligible age range (5–10 years) at each school. Given the manual nature of the process, a **simple manual selection method** was employed, where names of all listed students were drawn randomly by the investigator. Those selected were checked for eligibility (**including a quick clinical assessment by the investigator who is a medical doctor**), and if found ineligible, replacements were drawn using the same random selection process.

We have revised the manuscript to explicitly mention the type of random sampling used.

**Revised text**: A **simple random sample** of children aged 5–10 years was selected from each school using a list of enrollees in grades matching the eligible age groups. **A simple manual selection method was applied, where names of all listed students were drawn randomly by the investigator. Those selected were checked for eligibility, and if found ineligible, replacements were drawn using the same random selection process."**

**Reviewer comment: The school sector was used as a proxy for socioeconomic class: How could this be? Was it extremely difficult to elicit and classify the socioeconomic status of the parents/guardians? Some middle and low socio-economic class parents who appreciate the benefits of good education strive against all odds to offer their children good education.**

**Response:** We thank the reviewer for this important comment. Given the local context in Sudan, it was extremely challenging to categorize socioeconomic status using standard classification methods. This decision was based on the following considerations:

1. Unstable and inconsistent household income: A large proportion of families in Sudan do not have stable or predictable income sources, making direct financial classification unreliable. Additionally, cultural norms discourage open disclosure of income, further complicating accurate socioeconomic categorization.
2. Rapid fluctuations in local currency value: The Sudanese economy experiences frequent and significant currency devaluation, mostly on a daily basis. This makes income-based classification impractical and highly unstable for research purposes.
3. Challenges in applying standard socioeconomic measures: From our previous research experience in Sudan, we encountered considerable difficulty in assigning socioeconomic status based on standard classification systems. These measures often fail to account for local economic realities and do not translate well into this setting.

For these reasons, we carefully considered and decided to use school sector (public vs. private) as a proxy for socioeconomic class. While we acknowledge that some families from lower-income backgrounds may prioritize private education, these represent infrequent incidences, and this remains the most practical and widely accepted proxy in research conducted in similar settings. We have added a clarification in the manuscript to ensure transparency on this methodological decision.

**Revised text**: The school sector (public vs. private) was used as a proxy for socioeconomic class. This decision was based on local challenges in Sudan, where household income is often unstable, culturally sensitive to disclose, and subject to rapid currency fluctuations, making direct classification impractical. Standard socioeconomic measures have also been difficult to apply in this context. While not absolute, school sector remains a practical and widely used proxy in similar research settings.

**Reviewer comment: Prior to data collection, a pen-and-paper questionnaire was sent to the children's parents or guardians, seeking their informed consent for their child's participation: How did the authors handle rejection because it is obvious they did not meet the parents to explain.**

**Response**: We thank the reviewer for this comment. However, handling rejection was not a methodological concern, as data collection was conducted only among children whose parents or guardians provided written informed consent. **Parents who did not return the consent form were simply not included in the study**. Written consent was sought through a pen-and-paper questionnaire, which is a widely accepted and ethically sound approach in school-based research. While we did not meet parents in person, this does not impact the study's validity, as **participation was entirely voluntary and limited to those who consented.**

**Reviewer comment: The authors estimated a sample size of 392 but distributed 1000 consent forms to parents/guardians of the primary school children. Out of which 506 were returned recruited in the study. What was the reason for sending out 1000 consent forms? It implies that selection of the pupils for the study was dependent on return of consent forms. It is also not clear how they arrived at the 1000 pupils given the forms.**

**Response:** We thank the reviewer for this comment. This point has been fully addressed in our previous response, where we clarified the rationale for distributing 1000 consent forms and the selection process based on returned forms.

**Reviewer comment: Table 1: Please change Character to variables.**

**Response**: This has been amended as requested.

**Reviewer comment: The total after each variable should be expunged since there is total beneath the headings.**

**Response:** We thank the reviewer for this comment. We have removed totals for variables that included the entire sample, as these are already reflected in the column headings. However, the totals after some variables have been retained because **not all variables have data for the full sample size** due to missing responses or subgroup analyses. We believe this is necessary to avoid potential confusion, ensure transparency, and indicate the actual number of observations available for each variable.

**Revised text**: We have removed totals for the relevant variables.

**Reviewer comment: The m in the table should be written in full or explained as a footnote.**

**Response:** Thank you for drawing our attention to this point. We have amended it.

**Revised text:** m amended to “months”

**Reviewer comment: Monthly income category: The options under this variable is not clear. Example, Have to debt and saving do not show monthly income.**

**Response:** We thank the reviewer for this insightful comment. The categories listed in the table reflect the exact wording used in the questionnaire, as they were designed to capture **perceived financial status rather than actual income figures.**

While we acknowledge that 'Insufficient' and 'Have to debt' may appear overlapping, there is an important distinction:

- 'Insufficient' refers to families whose income does not fully cover their monthly expenses but does not necessarily mean they resort to borrowing. Some may adjust their spending or rely on irregular sources of support.
- 'Have to debt' specifically captures households that must borrow money regularly to meet basic financial needs, distinguishing them from those who simply find their income tight but manage without debt.

Since these responses **reflect parents' subjective financial perceptions**, we have kept the original wording to ensure the integrity of the data collected. However, we have clarified this distinction in the manuscript to improve transparency.

**Revised text:** Have to debt (must borrow to cover needs). Insufficient (struggling but no debt)

**Reviewer comment: School meal availability/intake: This is not clear. Availability and intake (consumption) are not the same.**

**Response:** “Availability” was removed to avoid confusion.

**Revised text**: “Availability” has been removed.

**Reviewer comment:** **Chi-squared or Fisher’s exact: Which one did the authors use?**

**Response**: We thank the reviewer for this comment. To clarify, we have added a footnote to Table 1 stating: *'Chi-squared test was used unless otherwise specified. Fisher’s exact test was applied where expected cell counts were <5.'*

**Revised text**: Chi-squared test was used unless otherwise specified. Fisher’s exact test was applied where expected cell counts were <5.

**Reviewer comment: Nutritional status and anthropometric measurements by school sector: What is the difference between nutritional status and anthropometric measurements? There are no weight and height measurements values in the table.**

**Response:** We thank the reviewer for this comment. We absolutely agree that Table 2 does not present raw anthropometric measurements (weight and height) but rather classifications based on Z-scores (WAZ, HAZ, and BAZ) and corresponding nutritional status categories. To ensure clarity, **we have revised the table title** to *'Nutritional Status and Growth Z-scores by School Sector'* to better reflect its content.

Revised text: Nutritional Status and Growth Z-scores by School Sector

**Reviewer comment: Figure 3: Pupils from public schools were 320. Out of this, 270 consume school meals and 44 did not giving a total of 314 instead of 320. Is there an explanation for this discrepancy?**

**Response:** We thank the reviewer for this observation. The discrepancy is due to missing responses from six participants in the school meal intake question, as **shown in Table 1**.

**Reviewer comment: Table 2 summarizes the stature and nutritional status: Is stature (height) not a parameter for assessing nutrition status? HAZ, WAZ, BMIAZ are all nutritional status indices.**

**Response:** We agree that stature (height) is a measure of **both growth and** nutritional status. HAZ, WAZ, and BAZ serve to standardize the classification of a child’s **growth and** nutritional status. This point has been addressed in a previous response.

**Reviewer comment: Growth is measured through nutritional status assessment. Using both the way it is seen in this study is somehow not clear. Increases or decreases in anthropometric parameters (weight, height, BMI) were not studied.**

**Response:** We have previously addressed this point, clarifying that nutritional status in children can be assessed through growth measurements, **and not vice versa**. Our study used **one-time** anthropometric measurements **rather than longitudinal tracking**, which is a standard approach in pediatric practice and research.

**Reviewer comment: Future research should aim to identify and investigate these factors: This better as Future research should aim to investigate and identify these factors.**

**Response:** This has been amended as requested.

**Revised text:** Future research should aim to investigate and identify these factors.

**Reviewer comment: School meals mentioned in this study did not specify if the meals were caregiver provided or government provided. Figure 3 shows that some children in public schools did not consume school meals.**

**Response:** We thank the reviewer for this comment. Our study assessed the effect of school meal intake **regardless of the source**. As stated in the manuscript ("No free or subsidized school meals are currently provided in schools in Sudan."), there are no government-provided school meals in Sudan.

**Reviewer comment: Ref 18 should be reviewed for journal title and appropriate punctuations.**

**Response**: This has been addressed. Thank you.

**Revised text**: Nat Hum Behav. 2017;1(5):1–7
