# Supplementary material for: Socioeconomic disparities in anthropometric status among primary school children: A potential association with school meals
Source: PLoS One. 2025 Apr 2;20(4):e0321289. doi: 10.1371/journal.pone.0321289 (PMC11964276; doi:10.1371/journal.pone.0321289)
Supplement: Attachment — Submitted filename: Reviewers comments on revised edition (Plos One 52592).docx [file pone.0321289.s004.docx]

**Reviewer’s comment:** I have read the authors’ response to the comments on growth and nutrition status. According to UNICEF ‘Weight gain is the most important sign that a child is healthy and is growing and developing well’ This is one of the basis for the suggestion to expunge growth.

<https://www.unicef.org/uganda/key-practice-monitoring-growth-and-development-child#:~:text=Weight%20gain%20is%20the%20most,if%20the%20child%20is%20overweight>.

Based on this reference, I would like to explain a bit further that when data on weight, height and BMI are obtained using a cross-sectional survey design, the single data are related to the child’s age and sex and compared to child growth standards as explained by the authors. This comparison yields anthropometric indices (weight-for-age and others) which are interpreted to showcase anthropometric status (a component of nutrition status) such as stunting, wasting, thinness and underweight. These can be used to identify children who are growing well. For the term growth to be used in a title of a manuscript, I expected to see weight gain calculated and not necessarily growth velocity/growth rate which are extension of growth assessment. Weight gain or weight loss as shown by UNICEF is the most important indicator of growth status. This is lacking in the manuscript. Besides, nutrition status assessment has 4 components (anthropometric, biochemical, clinical and dietary components). In assessment of nutrition status of individuals, at least 2 components should be used otherwise the specific component assessed (in this case anthropometric status) should be named and not the broad heading (nutritional status) despite the fact that some journals have published them. In the study under discourse, the authors used only anthropometry (one out of four) and therefore should use the term (anthropometric status) instead of nutrition status (for specificity). Again, their use of growth and nutrition status sort of separated growth from nutrition status which is correct because through out the study, they did not show weight status (gain or loss) or linear growth but compared the current anthropometric measurements as obtained cross sectionally with age and sex and interpreted them in line with child growth standards. Comparing anthropometric indices (HAZ, WAZ, BMIAZ, WHZ) with child growth standards would not yield data on growth status (gain or loss) but anthropometric status as normal, retarded (stunting, underweight, thinness and wasting) or above normal (overweight, obesity, too tall). Granted that some published articles may have used this same title, it does not seem to be correct. Based on this, I would suggest a rephrase of the title as Socioeconomic Disparities in Anthropometric Status Among Primary School Children in Central Sudan: A Potential Association with School Meals. This makes the title very specific to what the authors studied. Anthropometric assessment alone cannot showcase nutrition status as explained above. This is because nutrition status is much more than anthropometric status.

A study published in PLOS ONE journal showed growth and nutritional status clearly and there is no doubt about its title. Please see Hamid Namaganda L, Andrews C, Wabwire-Mangen F, Peterson S, Forssberg H, Kakooza-Mwesige A (2023) Nutritional status and growth of children and adolescents with and without cerebral palsy in eastern Uganda: A longitudinal comparative analysis. PLOS Glob Public Health 3(6): e0001241. ttps://doi.org/10.1371/journal.pgph.0001241

The authors may also wish to look at this article for growth assessment: Zoleko-Manego R, Mischlinger J, Dejon-Agobé JC, Basra A, Mackanga JR, Akerey Diop D, et al. (2021) Birth weight, growth, nutritional status and mortality of infants from Lambaréné and Fougamou in Gabon in their first year of life. PLoS ONE 16(2): e0246694. https://doi.org/10.1371/journal.pone.0246694.

Response: We sincerely appreciate the reviewer's comment. However, we respectfully disagree with the suggestion to remove 'growth' from the title for the following reasons:
• Our study, certainly, assessed growth using standard anthropometric measures:
We measured height, weight, and BMI and plotted them on established growth charts, which is the standard method for assessing one-point growth status in children.

**Reviewer’s response:** When this is done, the outcome is anthropometric status, one aspect of nutrition status assessment.

o This is the same approach used in clinical practice and epidemiological studies to assess child growth (<https://www.cdc.gov/growthcharts/background.htm>).

**Reviewer’s response:** Actually, it is to assess anthropometric status an aspect of nutrition status assessment as shown in the result section of this study.
• Nutritional status was assessed as a reflection of growth assessment:

**Reviewer’s response:** Anthropometric status and not nutrition status (one component out of 4 was assessed)
o We could not have assessed nutritional status without first assessing growth through these standard anthropometric indices.

**Reviewer’s response:** If you assessed growth, what was the outcome of your assessment? No table showed this.
o Both assessments were conducted at a single point in time, aligning with widely accepted methodologies (<https://www.ncbi.nlm.nih.gov/books/NBK537315>).

• Cross-sectional (one-off) growth assessments are an established method in child health research:

**Reviewer’s response:** I would rather say that anthropometric assessments are.

o The CDC growth charts, one of the most widely used references, were developed using this exact approach—measuring different children’s growth at different ages rather than tracking growth velocity over time.

**Reviewer’s response:** The emphasis is far from growth velocity.
o It is common practice in both clinical and public health research to use single-time-point growth assessments to evaluate children's health status (<https://pubmed.ncbi.nlm.nih.gov/12043359>).

**Reviewer’s response:** No doubt about this but it requires further assessment beyond what anthropometry reveals.
• We used 'growth charts,' not 'nutritional status charts.'

**Reviewer’s response:** There is no thing as nutritional status charts but child growth charts which reveal anthropometric status, a component of nutrition status.
o The charts and Z-score standards we used are called 'growth charts,' not 'nutritional status charts.'

o Removing 'growth' would inaccurately reflect the standard terminology used in growth and nutritional research (<https://www.who.int/tools/child-growth-standards/standards>).

**Reviewer’s response:** No, studies should report specifically what they revealed. This study was on anthropometric status of children using anthropometric indices (weight-for-age, height-for-age and BMI-for-age Z scores).
• Title relevance and study visibility:
o Keeping 'growth' in the title is essential for clarity, accuracy, and search visibility in scientific literature.

**Reviewer’s response:** Only if the authors assessed growth. They assessed anthropometric status using anthropometric indices derived from anthropometric measurements specific to age and sex.
o It correctly represents the study’s focus and ensures it is easily identified by relevant readers and researchers.
• Clarification on what we did not assess:
o We did not assess longitudinal growth velocity (growth rate over time), which seems to be the reviewer's concern.

**Reviewer’s response:** Of course, no data showed this. The issue is growth status and not growth rate. There is no way this study would show growth rate since it is design is cross sectional.
o However, cross-sectional growth assessments remain the primary method for evaluating child growth and nutritional status in epidemiological studies (<https://www.cdc.gov/growth-chart-training/hcp/using-growth-charts/who-methodology.html>).

**Reviewer’s response:** Cross-sectional growth assessments: the correct phrase is: cross sectional anthropometric assessment using WHO child growth standards. These standards relate anthropometric measurements/calculations (weight, height, BMI) to the age and sex of healthy children in the populations to identify those with deficits or excesses. It is not enough to assess nutrition status with only one component. It may be allowed but it is not appropriate to use nutrition status when this is done.
Given these points, we respectfully request that the reviewer reconsider this suggestion and allow us to retain 'growth' in the title and relevant sections.

**Reviewer’s response:** I am not convinced that the authors assessed growth of the children. They assessed anthropometric status by taking weight and height measurements and calculating BMI in a cross sectional study. They used child growth standards specific to age and sex to evaluate these parameters and arrived at the anthropometric status of the children showcasing their Z scores. The result section showed these clearly.

Reviewer comment: Abstract: The method section of the abstract is not detailed enough to show how the objective was achieved. Was comparison of the Z scores between private and public schools the only assessment conducted? The study design, sample size and sampling techniques used are missing. Which data collection methods were employed? How were the data analysed? The title showed that the authors also assessed growth. How was this conducted?
Response: We thank the reviewer for her valuable comments. We have revised the abstract to provide additional methodological details, including the study design, sample size, sampling method, data collection approach, and statistical analyses.
Regarding the reviewer's last point, we have already clarified in our previous response that growth was assessed using standard anthropometric measures (height, weight, and BMI), which were plotted on WHO growth charts. This remains the standard approach for evaluating growth at a single point in time in both clinical and epidemiological settings.

**Reviewer’s response:** What do the authors have as outcome of using standard anthropometric measures (height, weight, and BMI), plotted on WHO growth charts? If we take it that growth is assessed using anthropometric indices, what do the authors mean by nutrition status then? Would it not be unnecessary repetition? Anthropometry alone cannot stand for nutrition status because it is only one out of 4 components of nutrition status.

We cannot have growth and at the same time nutrition status in the same title where growth was not assessed to show gain or loss but growth parameters were related to age and sex to arrive at anthropometric indices and thus status. I am not satisfied with the explanation because the specific term assessed is missing in the title. Article titles should be direct, accurate and appropriate, precise and should not be misleading.

**Reviewer’s response:** How the weight and height values were converted into Z-scores is missing in the manuscript. How did the authors obtain BMI data before converting to Z scores? These details are necessary.

As discussed in our previous response, our study followed this standard cross-sectional methodology, making it an appropriate and widely accepted approach. We hope this clarification addresses the reviewer’s concern.

**Reviewer’s response:** ‘baseline and endline measurements reflects growth velocity assessment (growth rate over time)’. Not always, it depends on what the authors want to achieve. When the weights are plotted, a graph is produced to reveal weight gain, loss or static weight. The authors assessed anthropometric status of the children using growth parameters of weight, height and BMI. These were compared to sex and age and the indices interpreted to yield anthropometric status which is but one component of nutrition status.

Abstract conclusion

**Reviewer’s response:** Public school children exhibit unfavorable growth and nutritional status: Does this sentence imply that growth and nutrition status are one word or can be used interchangeably? There is no table or graph showing the growth status to be precise.

Revised text: We have reviewed the manuscript to ensure consistent usage of these terms following standard scientific conventions.
**Reviewer’s response:** All the anthropometric indices are written as compound words using hyphens, please.

**Reviewer’s response:** The use of the word around in ‘around 90000 primary school children’ showed approximation. Is it that the authors could not assess school registers to arrive at the exact population?

Revised text: A simple random sample of children aged 5–10 years was selected from each school using a list of enrollees in grades matching the eligible age groups. A simple manual selection method was applied, where names of all listed students were drawn randomly by the investigator. Those selected were checked for eligibility, and if found ineligible, replacements were drawn using the same random selection process."

**Reviewer’s response:** This is fine but it is obvious the authors do not want to disclose the number selected per school. It would have been good to see how the 1000 consent forms were distributed among the 10 schools.

**Reviewer’s response:** I would want to believe that the entire 10 schools had population that represented them well.

**Reviewer’s response:** Table 1: Please indicate that the ages are in years.

**Reviewer’s response:** Table 2: The reason for using Nutritional Status and Growth Z-scores in the same title is not clear.
**Reviewer’s response:** Title of Table 2 should be adjusted to: Mean Growth Z-scores and anthropometric status of the children.
