# Supplementary material for: Socioeconomic disparities in anthropometric status among primary school children: A potential association with school meals
Source: PLoS One. 2025 Apr 2;20(4):e0321289. doi: 10.1371/journal.pone.0321289 (PMC11964276; doi:10.1371/journal.pone.0321289)
Supplement: Attachment — Submitted filename: response_to_reviewers_auresp_2.docx [file pone.0321289.s005.docx]

# Responses to reviewers’ comments

## Reviewer #1

**Comment**: The paper seems too lengthy and can be cut short if this does not impact the overall structure and findings of this pieace of research

**Response**: We thank the reviewer for this suggestion. However, as no specific sections were specified for reduction, and the manuscript remains within the journal’s word limit, we believe maintaining the current length is necessary to preserve the clarity and completeness of our report.

## Reviewer #2

**Comment (all addressing same point):** I have read the authors’ response to the comments on growth and nutrition status. According to UNICEF ‘Weight gain is the most important sign that a child is healthy and is growing and developing well’ This is one of the basis for the suggestion to expunge growth.

https://www.unicef.org/uganda/key-practice-monitoring-growth-and-development-child#:~:text=Weight%20gain%20is%20the%20most,if%20the%20child%20is%20overweight.

Based on this reference, I would like to explain a bit further that when data on weight, height and BMI are obtained using a cross-sectional survey design, the single data are related to the child’s age and sex and compared to child growth standards as explained by the authors. This comparison yields anthropometric indices (weight-for-age and others) which are interpreted to showcase anthropometric status (a component of nutrition status) such as stunting, wasting, thinness and underweight. These can be used to identify children who are growing well. For the term growth to be used in a title of a manuscript, I expected to see weight gain calculated and not necessarily growth velocity/growth rate which are extension of growth assessment. Weight gain or weight loss as shown by UNICEF is the most important indicator of growth status. This is lacking in the manuscript. Besides, nutrition status assessment has 4 components (anthropometric, biochemical, clinical and dietary components). In assessment of nutrition status of individuals, at least 2 components should be used otherwise the specific component assessed (in this case anthropometric status) should be named and not the broad heading (nutritional status) despite the fact that some journals have published them. In the study under discourse, the authors used only anthropometry (one out of four) and therefore should use the term (anthropometric status) instead of nutrition status (for specificity). Again, their use of growth and nutrition status sort of separated growth from nutrition status which is correct because through out the study, they did not show weight status (gain or loss) or linear growth but compared the current anthropometric measurements as obtained cross sectionally with age and sex and interpreted them in line with child growth standards. Comparing anthropometric indices (HAZ, WAZ, BMIAZ, WHZ) with child growth standards would not yield data on growth status (gain or loss) but anthropometric status as normal, retarded (stunting, underweight, thinness and wasting) or above normal (overweight, obesity, too tall). Granted that some published articles may have used this same title, it does not seem to be correct. Based on this, I would suggest a rephrase of the title as Socioeconomic Disparities in Anthropometric Status Among Primary School Children in Central Sudan: A Potential Association with School Meals. This makes the title very specific to what the authors studied. Anthropometric assessment alone cannot showcase nutrition status as explained above. This is because nutrition status is much more than anthropometric status.

A study published in PLOS ONE journal showed growth and nutritional status clearly and there is no doubt about its title. Please see Hamid Namaganda L, Andrews C, Wabwire-Mangen F, Peterson S, Forssberg H, Kakooza-Mwesige A (2023) Nutritional status and growth of children and adolescents with and without cerebral palsy in eastern Uganda: A longitudinal comparative analysis. PLOS Glob Public Health 3(6): e0001241. ttps://doi.org/10.1371/journal.pgph.0001241

The authors may also wish to look at this article for growth assessment: Zoleko-Manego R, Mischlinger J, Dejon-Agobé JC, Basra A, Mackanga JR, Akerey Diop D, et al. (2021) Birth weight, growth, nutritional status and mortality of infants from Lambaréné and Fougamou in Gabon in their first year of life. PLoS ONE 16(2): e0246694. <https://doi.org/10.1371/journal.pone.0246694>.

When this is done, the outcome is anthropometric status, one aspect of nutrition status assessment.

Actually, it is to assess anthropometric status an aspect of nutrition status assessment as shown in the result section of this study.

Anthropometric status and not nutrition status (one component out of 4 was assessed).

If you assessed growth, what was the outcome of your assessment? No table showed this.

I would rather say that anthropometric assessments are.

The emphasis is far from growth velocity.

No doubt about this but it requires further assessment beyond what anthropometry reveals.

There is no thing as nutritional status charts but child growth charts which reveal anthropometric status, a component of nutrition status.

No, studies should report specifically what they revealed. This study was on anthropometric status of children using anthropometric indices (weight-for-age, height-for-age and BMI-for-age Z scores).

Only if the authors assessed growth. They assessed anthropometric status using anthropometric indices derived from anthropometric measurements specific to age and sex.

Of course, no data showed this. The issue is growth status and not growth rate. There is no way this study would show growth rate since it is design is cross sectional.

Cross-sectional growth assessments: the correct phrase is: cross sectional anthropometric assessment using WHO child growth standards. These standards relate anthropometric measurements/calculations (weight, height, BMI) to the age and sex of healthy children in the populations to identify those with deficits or excesses. It is not enough to assess nutrition status with only one component. It may be allowed but it is not appropriate to use nutrition status when this is done.

 I am not convinced that the authors assessed growth of the children. They assessed anthropometric status by taking weight and height measurements and calculating BMI in a cross sectional study. They used child growth standards specific to age and sex to evaluate these parameters and arrived at the anthropometric status of the children showcasing their Z scores. The result section showed these clearly.

What do the authors have as outcome of using standard anthropometric measures (height, weight, and BMI), plotted on WHO growth charts? If we take it that growth is assessed using anthropometric indices, what do the authors mean by nutrition status then? Would it not be unnecessary repetition? Anthropometry alone cannot stand for nutrition status because it is only one out of 4 components of nutrition status.

We cannot have growth and at the same time nutrition status in the same title where growth was not assessed to show gain or loss but growth parameters were related to age and sex to arrive at anthropometric indices and thus status. I am not satisfied with the explanation because the specific term assessed is missing in the title. Article titles should be direct, accurate and appropriate, precise and should not be misleading.

‘Baseline and endline measurements reflects growth velocity assessment (growth rate over time)’. Not always, it depends on what the authors want to achieve. When the weights are plotted, a graph is produced to reveal weight gain, loss or static weight. The authors assessed anthropometric status of the children using growth parameters of weight, height and BMI. These were compared to sex and age and the indices interpreted to yield anthropometric status which is but one component of nutrition status.

Public school children exhibit unfavorable growth and nutritional status: Does this sentence imply that growth and nutrition status are one word or can be used interchangeably? There is no table or graph showing the growth status to be precise.

**Response**: We appreciate your comment. The title has been changed as per the reviewer’s comment.

**Comment**: The use of the word around in ‘around 90000 primary school children’ showed approximation. Is it that the authors could not assess school registers to arrive at the exact population?

**Response**: We thank the reviewer for this comment. The school registries do not contain precise records of student enrollment. Therefore, we relied on the official estimates provided by the State Ministry of Education, which represent the best available data for the primary school population.

**Comment**: This is fine but it is obvious the authors do not want to disclose the number selected per school. It would have been good to see how the 1000 consent forms were distributed among the 10 schools.

**Response**: We thank the reviewer for this comment. Unfortunately, we do not have a precise record of the distribution of consent forms across the 10 schools. However, we believe this does not significantly impact the methodological rigor of the selection process.

**Comment**: I would want to believe that the entire 10 schools had population that represented them well.

**Response**: We thank the reviewer for this comment. As no specific change or recommendation was provided, we believe no revisions to the manuscript are necessary at this stage.

**Comment**: Table 1. Please indicate that the ages are in years.

**Response**: Thank you, this has been indicated.

**Comment**: Table 2: The reason for using Nutritional Status and Growth Z-scores in the same title is not clear.

**Response**: We appreciate the reviewer’s feedback. The title was changed to “Mean Growth Z-scores and anthropometric status of the children”.
